# Development of a 3-day manufacturing method to generate CD19-CD20-CD22 trispecific CAR T-cells from whole blood

**DOI:** 10.1186/s12967-026-08306-8

**Published:** 2026-07-15

**Authors:** Isabella Vignola, Michaela Prochazkova, Lipei Shao, Tatyana Fuksenko, Sarmila Sarkar, Hong Lei, Jianjian Jin, Ying Xiong, Yanping Xie, Ibeawuchi Oparaocha, Oxana Slessareva, Megan Forrest, Rimas Orentas, Zhongyu Zhu, Boro Dropulic, Ping Jin, Robert P. Somerville, David F. Stroncek, Hannah W. Song, Steven L. Highfill

**Affiliations:** 1 Center for Cellular Engineering, Department of Transfusion Medicine, National Institutes of Health, Bethesda, MD USA; 2 Caring Cross, Gaithersburg, MD USA; 3 Department Biochemistry and Molecular Biology, The Johns Hopkins Bloomberg School of Public Health, Baltimore, MD USA

**Keywords:** Chimeric antigen receptor, Whole blood, Negative selection, Cell manufacturing, Cellular therapy

## Abstract

**Background:**

Chimeric antigen receptor (CAR) T-cell therapy has transformed the treatment landscape for many hematological malignancies. However, high relapse rates and limited accessibility remain significant challenges. We developed a 3-day streamlined process to address these limitations.

**Methods:**

T-cells were isolated from whole blood collected from healthy donors via an automated density gradient separation that included a T-isopure™ antibody cocktail, which isolates T-cells through negative selection. T-cells were activated then transduced with a lentiviral vector encoding a trispecific CAR. T-cells were cultured in G-Rex vessels and harvested at day 3 or day 7 for analysis. CAR expression and T-cell phenotype were assessed by flow cytometry and gene expression analysis. Functional activity was evaluated by measuring cytotoxicity and cytokine secretion following co-culture with target cell lines.

**Results:**

The T-isopure isolation enriched CD3^+^ T-cells in whole blood from 18.9% to 88.5% of CD45^+^ cells, with a mean recovery of 40.6%. RBCs were depleted with ≥ 99% efficiency, with monocytes and NK cells comprising the bulk of remaining CD45^+^ cells. The 3-day manufacturing process produced T-cells with > 95% viability, 53% transduction efficiency, and vector copy number < 3 copies/cell. Phenotypic analysis revealed a high proportion of stem/central memory T-cells at both timepoints, with no significant differences observed. Cytotoxicity assays demonstrated strong and sustained killing of NALM6 tumor cells, comparable between both products. Gene expression profiling indicated that day 3 products were less differentiated, exhibiting a memory-like phenotype and reduced inflammatory signaling, further supported by protein analysis of culture supernatants.

**Conclusion:**

This study establishes a rapid, GMP-compliant method for manufacturing polyfunctional, CAR T-cells directly from whole blood. The workflow outlined here achieved a potent, phenotypically favorable CAR T-cell product without compromising viability or cytotoxic function. Compared to the standard 7-day method, the 3-day approach resulted in expression of genes associated with a more stem-like phenotype while reducing manufacturing time and cost. This method may provide a practical alternative for decentralized CAR T-cell manufacturing, particularly in resource-limited settings.

**Supplementary Information:**

The online version contains supplementary material available at 10.1186/s12967-026-08306-8.

## Introduction

Chimeric antigen receptor (CAR) T-cells are genetically modified T lymphocytes engineered to express receptors that target specific antigens [1, 2]. Since the first FDA approval in 2017, seven CAR T-cell therapies have been approved for the treatment of hematological malignancies [3, 4]. CD19- and BCMA- directed CAR T-cell products have demonstrated substantial clinical efficacy, achieving complete response rates of approximately 40–60% in patients who had relapsed after multiple lines of therapy [2, 5–8]. This success has fueled the rapid expansion and diversification of the field, with CAR T-cell therapies now being investigated for solid tumors, autoimmune diseases, and certain infectious diseases [3, 9].

Despite this progress, widespread clinical implementation remains limited by several major challenges. Relapse rates are high, reaching up to 70% within 12–24 months for patients with B-ALL and over 80% for patients with multiple myeloma [5–8]. This high relapse rate underscores the need for next-generation strategies, including trispecific CAR constructs capable of simultaneously targeting multiple antigens to mitigate antigen escape and reduce disease recurrence. It has been suggested that relapse may be further reduced if the CAR T-cell product is composed of a higher proportion of naïve T-cells with the capacity for long-term persistence [10, 11]. Another key barrier is cost, which is largely driven by the complex workflows required for manufacturing. A 2018 analysis reported that treatment with tisagenlecleucel averaged $510,963 per patient [12], reflecting the expense of viral vector production, maintenance of GMP-compliant facilities, and the operation of specialized equipment and reagents.

All commercially approved CAR T-cell products use autologous leukapheresis as a starting product, which must be collected in specialized centers with dedicated equipment and trained medical staff. We have previously shown that whole blood starting material may be suitable for CAR T-cell manufacturing and offers a more accessible alternative for T-cell collection [13], especially in decentralized or low-resource settings. Whole blood is not commonly used because of the lower frequency and total number of T-cells obtained, and higher red blood cell content than in leukapheresis products. However, a lack of studies detailing GMP compliant methods for T-cell isolation from whole blood and the relatively long culture duration used to produce CAR T-cells remain key hurdles for implementation, although several recent studies indicate promising results [13–17]. Recent work has further shown that trispecific duoCAR-T cells can overcome antigen escape and eliminate heterogeneous B-cell tumors in preclinical models, underscoring the potential of continued innovation to address current limitations in CAR T-cell therapy [18].

To address these limitations, we developed a novel 3-Day manufacturing process starting with whole blood that generates a CAR T-cell product using a previously described trispecific CD19-CD20-CD22 CAR lentiviral vector construct [18, 19]. While the trispecific CAR and its favorable properties have been reported previously, the innovation of this study lies in the development of the shortened manufacturing process. T-cells are purified via negative selection using a customized GMP T-isopure antibody cocktail and an automated density gradient separation. Following isolation, cells underwent activation, transduction, and a brief expansion phase to preserve a more naïve CAR T-cell phenotype. We demonstrate that this cost-effective, accelerated manufacturing approach produces a more naïve yet functionally competent T-cell drug product with enhanced potential for long-term persistence in patients.

## Methods

### Collection of peripheral blood

All healthy subjects and patient provided written informed consent prior to participation. Peripheral blood samples were obtained from 11 healthy donors and one patient with pancreatic cancer, either collected at the Department of Transfusion Medicine, National Institute of Health (NIH, Bethesda MD) or purchased from CGT Global (Folsom CA). Healthy donors from the NIH (*N* = 7) were collected in Tricitrasol (Citra Labs) and processed on the same day. Healthy donor samples (*N* = 4) and a patient donor (*N* = 1) from CGT Global were collected in sodium heparin, shipped overnight at room temperature, and processed the following day. Donor characteristics are listed in Supplemental Table 1.

### T-cell purification via negative selection

Whole blood samples were first analyzed on a XN-20 Hematology Analyzer (Sysmex) for a complete blood count (CBC) and a FACSLyric flow cytometer (BD Biosciences) for phenotyping. T-cells were purified via negative selection using the T-isopure antibody cocktail (Caring Cross) in combination with Ficoll-Paque (GE Healthcare) density gradient separation performed on the Sepax 2 RM (Cytiva).

Prior to processing, whole blood samples were volume-reduced to ≤ 110 mL. If this was not possible due to cell pellet volume, the samples were reduced to ≤ 220 mL and split into two aliquots for processing. The T-isopure antibody cocktail was added at a ratio of 2 µL per 1 mL of whole blood and incubated at room temperature for 20 min on a rotator to facilitate antibody binding. Following incubation, density gradient separation was performed using the NeatCell v318 program on the Sepax 2 RM (Cytiva) with Ficoll-Paque (GE Healthcare). Kits were prepared according to the manufacturer’s instructions, using Plasmalyte-A (Baxter) as a wash buffer with two wash cycles and high purity setting “on.” Post-isolation, purity and recovery of T-cells were evaluated using the Sysmex and flow cytometry. Isolated T-cells were then prepared for culture and expansion.

### Manufacturing CAR T-cells

To directly compare CAR T-cell products from day 3 vs. day 7, isolated T-cells were seeded into one culture vessel and subsequently split in half on day 3 as described below, with half the culture volume being harvested and cryopreserved on day 3 and the other half remaining in culture until day 7. Cultures were initiated with 50 million CD3^+^ T-cells seeded into a G-Rex 10 M vessel (Wilson Wolf). On day 0, in 10 mL of TexMACS media (Miltenyi) supplemented with 3% heat-inactivated AB serum (Valley Biomedical) and 200 IU/mL of IL-2 (Clinigen). TransAct (Miltenyi) was added at a concentration of 1 mL TransAct per 17.5 mL total culture volume (571 µL per 10mL culture). Cultures were incubated at 37 °C in a humidified incubator with 5% CO_2_.

A trispecific CD19-CD20-CD22 CAR (CX170, Caring Cross, Gaithersburg, MD), delivered via lentiviral vector encoding a tandem CAR targeting CD19-CD20 and a separate CAR against CD22 was used [18–20]. Unless otherwise noted, transduction was performed at a multiplicity of infection (MOI) of 18.2. On day 1, 3.5 mL of spent media was removed from the supernatant, 3.5 mL of diluted vector was added, and the cells were incubated with vector for 24-hours. On day 2 (i.e., 24 h after transduction), 90 mL of fresh media was added to each G-Rex to dilute the lentiviral vector (no wash), bringing the total volume to 100 mL.

On day 3, 90 mL of spent media was removed from the supernatant, and cells were resuspended and assessed for total count and viability via AOPI (Cellometer, Revvity), vector copy number (VCN), and transduction efficiency via flow cytometry. Half of the product was harvested and cryopreserved using a 1:1 mixture of CryoStor-CS10 (BioLife Solutions) and Plasmalyte-A supplemented with 4% human serum albumin (HSA; Baxalta). The remaining cells were resuspended in fresh media to a final volume of 100 mL and returned to the incubator. On day 7, the same assessments were repeated using the same procedure as on day 3. Cells were cryopreserved as described for day 3 and stored in liquid nitrogen for functional assays.

### Flow cytometry

Cells were resuspended in PBS (Baxter) supplemented with 0.5% HSA (Baxalta) and stained for 30 min at 4 °C. Cells were washed once, resuspended at approximately 1 × 10^6^ cells/mL in FACS buffer (PBS, 2% FBS, 2 mM EDTA) and run on either a FACSLyric (BD Biosciences) or Cytoflex flow cytometer (Beckman Coulter). Flow analysis was performed in FCS Express 7.20.0023 (DeNovo Software). Representative gating strategies can be found in Supplemental Figs. 1 and 2 for determining cell population frequencies and T-cell stemness, respectively. Antibodies used for flow cytometry are listed in Table S2.

### In vitro cytotoxicity assay

Transduced cells cryopreserved on day 3 and day 7, as well as untransduced controls (UNT) on day 7, and green fluorescent protein (GFP) labeled NALM6 tumor cells were thawed and allowed to rest overnight in MEM-Eagle medium with 10% fetal bovine serum (FBS). The next day, T-cells were co-cultured with 5 × 10^4^ NALM6 tumor cells at effector to target ratios of 1:1, 2:1, and 5:1 in triplicate in a flat bottom 96 well plate. Effector cell concentrations of day 3 and day 7 products were calculated based on transduction efficiency (%G4S^+^) as measured on day 7, as expression kinetics showed that not all cells expressed CAR as early as day 3. After setup, the plate was placed in a humidified incubator at 37 °C and 5% CO_2_, and remaining tumor was quantified at 48, 96, and 144 h by either IncuCyte SX5 Live-Cell Analysis System (Sartorious) or flow cytometry using CountBright absolute counting beads (Invitrogen) per manufacturer’s instructions. Percent cytotoxicity was then calculated by the percent difference between the total tumor area quantified via the IncuCyte system or total tumor cell number normalized to number of counting beads of the tumor-only well and the treated wells. Every two days, 100 µL of supernatant was removed, and 5 × 10^4^ fresh NALM6 cells were added in equivalent volume. Supernatants were saved at -80 °C for cytokine analysis.

### Cytokine analysis

Cryopreserved cell culture supernatants were thawed on ice and analyzed straight (i.e., undiluted) using the Bio-Plex 200 suspension multiplex system (Bio-Rad) with the Bio-Plex Human Cytokine Screening Panel, which includes 48 cytokine and chemokine cell signaling molecules (β-NGF, CTACK, Eotaxin, FGF basic, G-CSF, GM-CSF, GRO-α, HGF, IFN-α2, IFN-γ, IL-1α, IL-1β, IL-1ra, IL-2, IL-2Ra, IL-3, IL-4, IL-5, IL-6, IL-7, IL-8, IL-9, IL-10, IL-12(p70), IL-12(p40), IL-13, IL-15, IL-16, IL-17 A, IL-18, IP-10, LIF, M-CSF, MCP-1(MCAF), MCP-3, MIF, MIG, MIP-1α, MIP-1β, PDGF-bb, RANTES, SCF, SCGF-β, SDF-1α, TNF-α, TNF-β, TRAIL, VEGF). Cytokine concentrations were determined using the BioPlex 200 reader and Bio-Plex Manager Software.

### Gene expression analysis

Total RNA was isolated from CAR T-cell products using the miRNeasy Mini Kit. RNA concentration and quality were assessed using the Nanodrop 8000 and Agilgent 2100 Bioanalyzer. A total of 100 ng of RNA was hybridized in solution with the NanoString nCounter CART panel. Hybridized samples were loaded onto cartridge, sealed, and processed according to the manufacturer’s instructions. RCC files containing raw gene counts were generated and imported into ROSALIND platform for normalization. Differential gene expression was analyzed using the *limma* package (|fold change| ≥ 2 & adjusted *p-*value < 0.05). Pathway scores were calculated by the *GSVA* package. All analyses were performed in the RStudio environment with customized scripts.

### VCN assay

Genomic DNA was isolated from cryopreserved cell pellet using the DNeasy Blood and Tissue Kit (Qiagen) according to the manufacturer’s instructions. Droplets were generated on the QX200 Automated Droplet Generator (AutoDG) system (Bio-Rad). PCR was performed on a Bio-Rad T100 Thermal Cycler, and the droplets were read using the QX Droplet Reader (Bio-Rad) and analyzed by QuantaSoft software. VCN was calculated as the ratio of transgene to reference copies per diploid genome.

### Statistics

GraphPad Prism 10 was used to generate the figures, statistical analysis was performed in RStudio. A p-value of < 0.05 was considered statistically significant.

## Results

### T-cell purification from whole blood

**Fig. 1:**
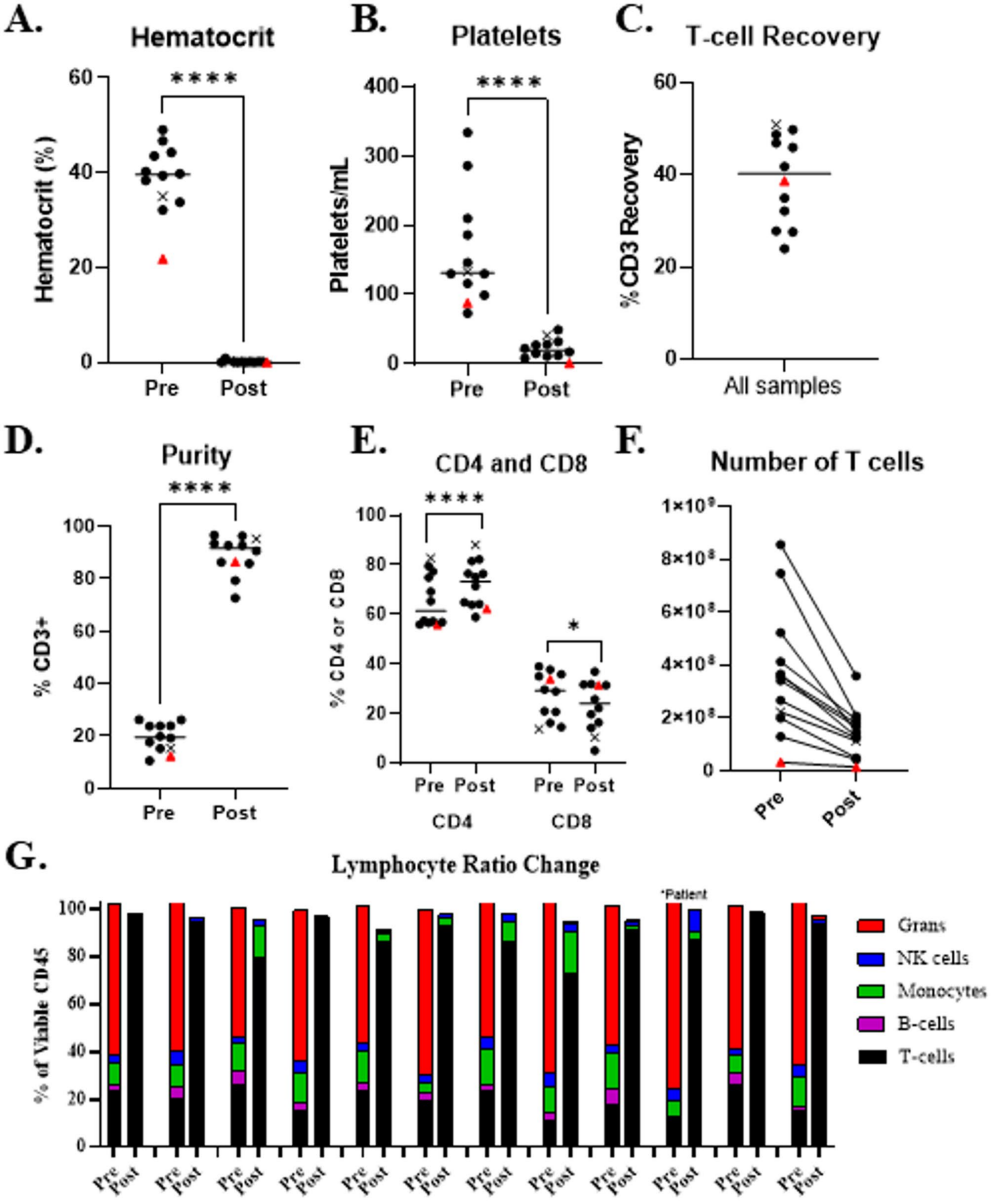
T-cell Isolation from Whole Blood Using T-isopure Negative Selection. **(A)** Hematocrit (%) before and after processing. **(B)** Absolute number of platelets per mL before and after processing. **(C)** CD3 T-cell recovery (%) after being processed. **(D)** T-cell purity (%) before and processing. CD3 gated from viable CD45^+^. **(E)** Frequency of CD4^+^ and CD8^+^ T-cells (%) before and after processing. **(F)** The initial and final absolute number of T-cells in each sample. **(G)** CD45^+^ population breakdown of granulocytes, natural killer cells, monocytes, B-cells, and T-cells before and after processing for each of the products tested. These populations were measured by flow cytometry and gated on singlets, viable, CD45^+^. In parts A, B, C, D, E, and F, the red triangle indicates the patient sample, the x indicates a manual Ficoll. **** = *p* < 0.0001, * = *p* < 0.05

Whole blood was collected from 11 healthy donors ranging in age from 25 to 79, with collection volumes ranging from 130 to 400 mL (mean, 272.5 mL) (Supp. Table 1). As expected, there was substantial variability between donors in whole blood composition. Hematocrit values ranged from 32.1% to 49.0% (mean, 40.2%)(Fig. 1A, Supplementary Table 1), and platelet counts ranged from 73 × 10^6^ to 334 × 10^6^ (mean, 167.5 × 10^6^ platelets/mL)(Fig. 1B, Supplementary Table 1).

T-cell purification was performed directly from whole blood using the T-isopure antibody cocktail. This cocktail targets non-T-cell WBC populations (CD19, CD14, CD11b, CD16) and crosslinks them to red blood cells (RBCs), facilitating their removal via standard density gradient separation. This reagent was designed to be utilized for the high RBC to WBC ratio in whole blood and is not compatible with apheresis unless it is supplemented with RBCs.

After isolation, the hematocrit significantly decreased by 99.7%, and the platelets by 84.3% (Fig. 1A, B). Flow cytometry was used to characterize WBC subpopulations (representative gating is shown in Supp Fig. 1). T-cell recovery averaged 40.6% following isolation, during which the target CD3^+^ T-cell population increased from an initial mean of 18.9% of the CD45^+^ WBC population to 88.5% post-isolation (Fig. 1C, D). Within the T-cell population, CD4^+^ T-cells increased from 66.7% to 73.1% post-isolation (*p* = 7.3 × 10⁻⁵), while CD8^+^ T-cells decreased from 26.1% to 22.4% (*p* = 0.022), indicating a modest yet statistically significant shift toward CD4^+^ phenotype post isolation (Fig. 1E). The absolute number of T-cells for the input material (pre-isolation) was variable (between 1.01 × 10^8^ and 7.65 × 10^8^) but was more consistent in the post-processed product (between 4.22 × 10^7^ and 3.47 × 10^8^) (Fig. 1F).

Non-T-cell CD45^+^ populations were significantly depleted following isolation. The largest cell population in the input material, CD15^+^ granulocytes, decreased from an average of 63.9% to approximately 0.4% (99.3% frequency reduction). CD19^+^ B-cells were reduced from an average of 3.4% to approximately 0.1% (92.0% frequency reduction). CD14^+^/CD16^+^ monocytes decreased from 10.6% to 4.6% on average (54.9% frequency reduction) and were the largest non-CD3^+^ population remaining after isolation. CD56^+^ NK cells decreased from an average of 4.4% to approximately 2.3%, (49.1% frequency reduction) (Fig. 1G). These data confirm effective isolation of CD3^+^ T-cells and removal of contaminating WBC cells, especially CD15^+^ granulocytes and CD19^+^ B-cells.

### CAR T-cell manufacturing and phenotyping

**Fig. 2:**
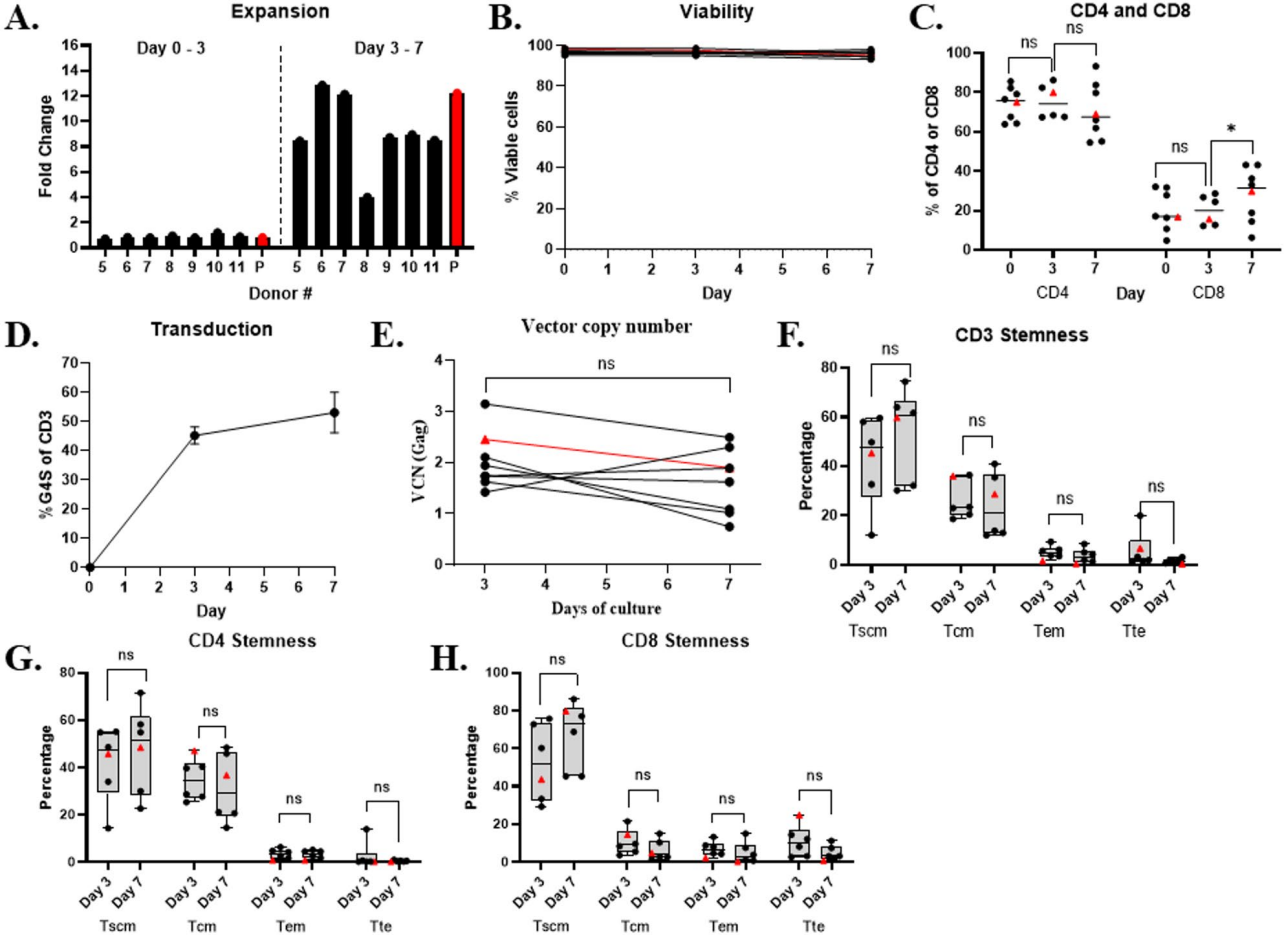
CAR T-cell Manufacturing. **(A)** Fold change expansion calculated by comparing the D3 to the D0 total cell number (left) or D7 to the D3 total cell number (right). P, patient sample. **(B)** Percent viability of cultures on days 0, 3, and 7. **(C)** Relative frequency of CD4^+^ and CD8^+^ T-cells on days 0, 3, and 7. **(D)** Expression of G4S determined by flow cytometry on days 0, 3, and 7. **(E)** Vector copy number on days 3 and 7. T-cell stemness (CD3, **F**; CD4, **G**; CD8, **H**), measured by flow cytometry with the different subsets defined as; Tscm/naive = CD62L^+^, CCR7^+^, CD45RA^+^; Tcm = CD62L^+^, CCR7^+^, CD45RA^−^; Tem = CD62L^−^, CCR7^−^, CD45RA^−^; Tte = CD62L^−^, CCR7^−^, CD45RA^+^, on days 3 and 7. Red triangle in the figure represent the patient sample

We wanted to optimize a short CAR-T manufacturing process of 3 days, which would let us stimulate the T-cells and allow for sufficient time for lentiviral transduction. Included in these experiments was a day 7 culture control, which had identical processing for days 0–3, but included a longer cell expansion period more representative of other standard CAR T-cell manufacturing procedures. Isolated T-cells were cultured in G-Rex 10 M flasks and activated with TransAct and IL-2, with a 24-hr lentiviral transduction on day 1. The 3-day culture period resulted in a slight decrease in cell number, with a mean fold change of 0.88 ± 0.15. From day 3 to day 7, cultures expanded by a mean fold change of 9.48 ± 2.9 (Fig. 2A). Viability remained consistently high during the entire culture period (day 3: 96.6 ± 0.99; day 7 95.7 ± 1.61) (Fig. 2B). A comparison of the CD4^+^ and CD8^+^ T-cell frequencies at day 3 and day 7 compared to day 0 show that over time, the average frequency of CD8^+^ T-cells appeared to rise, consistent with the rapid activation and expansion kinetics of this subset after antigen stimulation [21]. CD8^+^ T-cells increased from 20.2% ± 7.4% on day 3 to 28.2% ± 13.6% on day 7 (mean difference: +9.2%, *p* = 0.03). On day 3, the CD4^+^ comprised an average of 75.4% ± 8.5% of the total T-cell population, decreasing slightly to 70.5% ± 14.0% by day 7 (mean difference: -6.1%, *p* = 0.1584) (Fig. 2C). Despite the statistically significant increase in CD8^+^ cells from day 3 to day 7, the CD4^+^ cells did not change in a statistically significant way.

Transduction efficiency was determined using an anti-G4S antibody that recognizes the linker portion of the CAR. Expression of the CAR by flow cytometry on day 3 had a mean transduction efficiency of 47.51% ± 4.6%, and on day 7 had a mean transduction efficiency of 52.4% ± 7.6% (Fig. 2D). Transduction efficiency on day 3, however was more difficult to assess due to the lack of a clear threshold for CAR-positive cells (Supplementary Fig. 2). Vector copy number (VCN) per cell averaged 2.02 ± 0.55 on day 3 and decreased to 1.63 ± 0.63 on day 7 (*p* = 0.15) (Fig. 2E).

Next, we evaluated T-cell differentiation surface phenotype for the day 3 versus the day 7 cultures. The T-cell subset phenotype was assessed using CD62L, CCR7 and CD45RA markers to define subsets as follows: Tscm, CCR7^+^CD45RA^+^CD62L^+^; Tcm, CCR7^+^CD45RA^−^CD62L^+^; Tem, CCR7^−^CD45RA^−^CD62L^−^; Tte, CCR7^−^CD45RA^+^CD62L^−^ (see Supplementary Fig. 3 for representative gating strategy). The frequency of Tscm increased from a mean of 43.0% on day 3 to 52.7% on day 7, though this difference was not statistically significant (*p* = 0.43). Tcm cells remained roughly the same (26.4% to 25.0%, *p* = 0.72), as did Tem cells (5.0 to 3.3, *p* = 0.23). Tte cells declined from 5.7% to 1.5% (*p* = 0.10) (Fig. 2F). Despite these shifts, none of the changes reached statistical significance. When subset phenotypes were evaluated separately within the CD4 and CD8 T-cell compartments, no statistically significant differences were observed between day 3 and day 7 cultures (Fig. 2G and H).

These findings demonstrate that the shortened 3-day CAR-T manufacturing process maintains high viability, transduction efficiency, and T-cell stemness.

### Functional assays: cytotoxicity and cytokine production

**Fig. 3:**
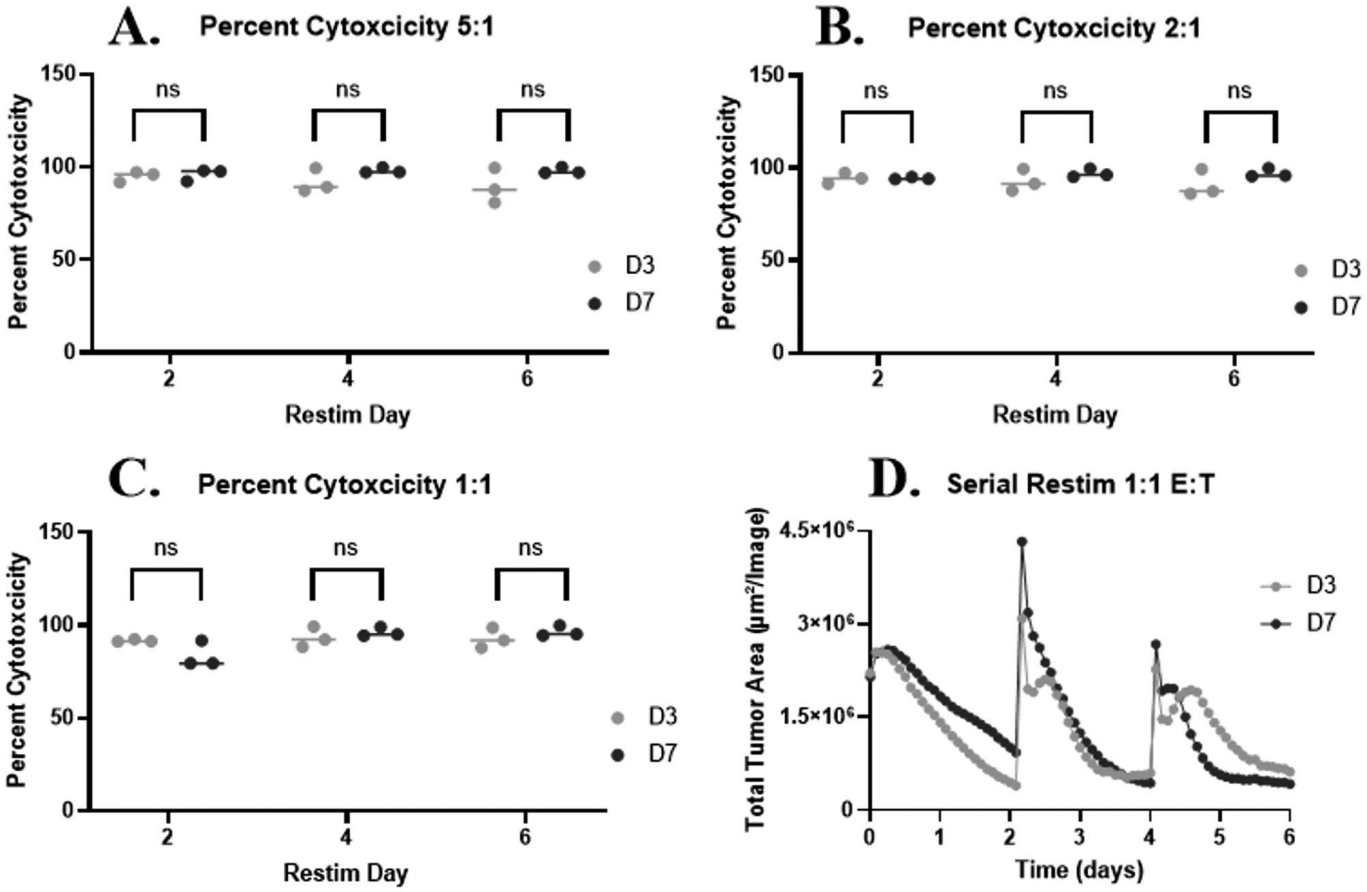
Day 3 (early) CAR products show comparable cytotoxicity to day 7 (late) products. Average percent cytotoxicity of the 3-day CAR, the 7-day CAR, and an untransduced control, when co-cultured with NALM6 tumor cells. Every 48 h, the cells were restimulated with additional NALM6 tumor cells to assess their ability to kill over time. **(A)** Percent cytotoxicity of CAR cells co-cultured with NALM6 cells at a 5:1 E: T ratio. The same patterns were also seen at E: T ratios of 2:1 **(B)** and 1:1 **(C)**. **(D)** Representative plot of tumor area during repeated stimulation

Cytotoxicity of day 3 and day 7 CAR T-cell products was evaluated using a tumor co-culture assay with GFP-labeled NALM6 tumor cells across effector-to-target (E: T) ratios of 5:1, 2:1, and 1:1. Every 48 h, fresh NALM6 tumor cells were added to measure cytotoxicity over time. At the 5:1 E: T ratio, the day 3 product showed average cytotoxicity of 95.0%, 92.0%, and 89.4% at restimulation on days 2, 4, and 6, respectively (Fig. 3A). The day 7 product showed average cytotoxicity of 96.0%, 98.0%, 97.9%. At the 2:1 E: T ratio, day 3 products demonstrated cytotoxicity of 94.5%, 93.0%, and 91.0%, while day 7 products had 94.5%, 97.1%, and 97.1% (Fig. 3B). At the lowest tested E: T ratio of 1:1, the day 3 product averaged 91.8%, 93.3%, and 92.9% compared to 83.6%, 96.2%, and 96.6% for the day 7 product (Fig. 3C). Overall, the two products demonstrated comparable cytotoxic activity across all timepoints and effector-to-target ratios (Fig. 3D). These findings indicate that 3-day CAR T-cell products retain potent and durable cytotoxic function equivalent to conventionally expanded products.

**Fig. 4:**
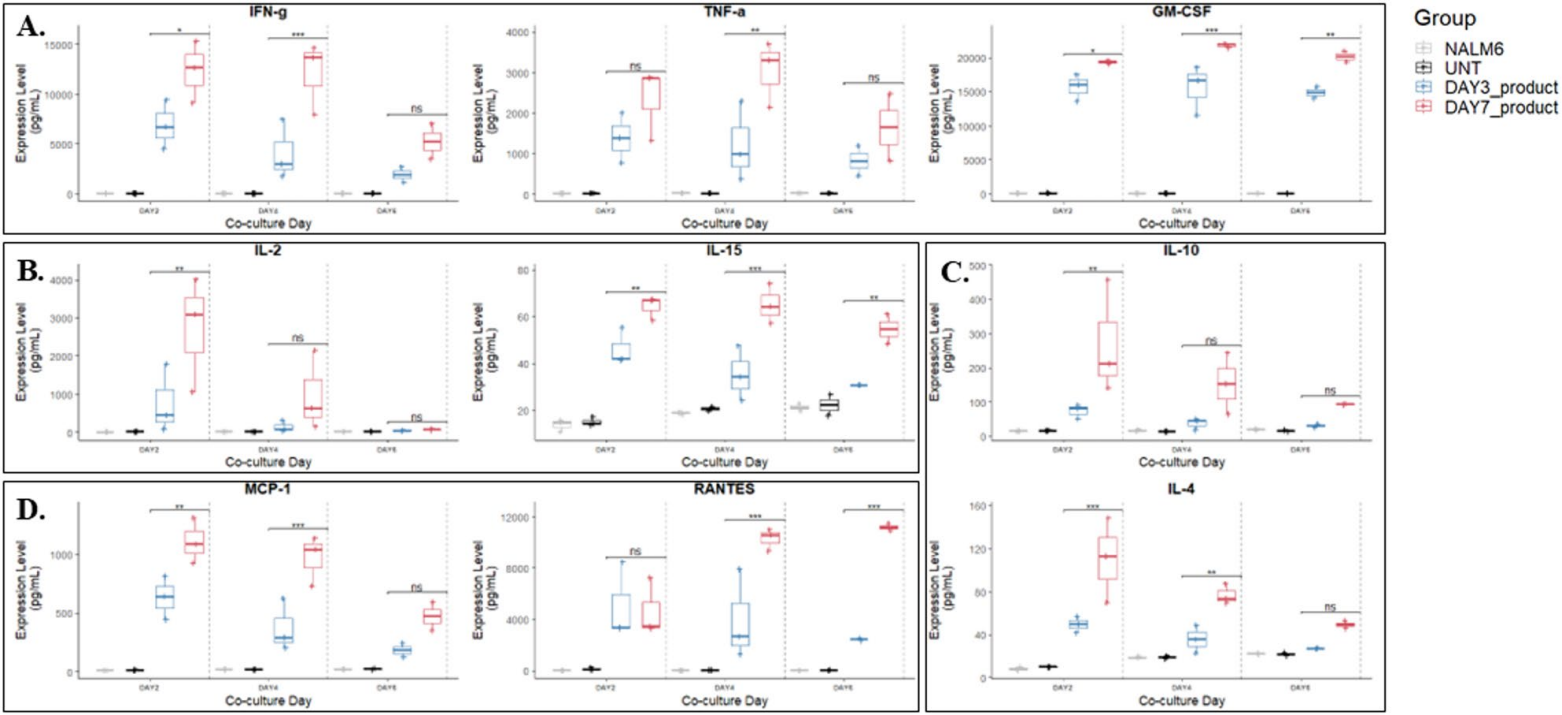
Cytokine profiling of the 3-day (early) versus the 7-day (late) CAR T-cell products. Cytokine secretion was measured following co-culture with target cells at day 2, 4, and 6. Data are shown as box-and-whisker plots with median, interquartile range, and individual donor variability. Significance was determined by two-way ANOVA with Tukey-adjusted pairwise contrasts. **(A)** Effector function was examined by measuring IFN-g, TNF-α, and GM-CSF. **(B)** Proliferation potential was examined by measuring IL-2 and IL-15 secretion. **(C)** T-cell regulation function was examined by measuring IL-10 and IL-4. **(D)** T-cell trafficking potential was examined by measuring the chemokines MCP-1 (CCL2) and RANTES (CCL5). *** = *p* < 0.001, ** = *p* < 0.01, * = *p* < 0.05, ns = *p* > 0.05

Upon stimulation with NALM6 cells, both day 3 and day 7 CAR T-cell products secreted a broad array of effector and pro-inflammatory cytokines compared to unstimulated controls (UNT) and NALM6-only wells. High levels of IFN-γ, TNF-α, and GM-CSF confirmed robust effector function in both products, with day 7 cells generally producing greater amounts (Fig. 4A). Cytokines supporting proliferation and persistence (IL-2 and IL-15) were also elevated after antigen stimulation, again trending higher in day 7 products (Fig. 4B). Regulatory cytokines (IL-4 and IL-10) were more abundant in day 7 products but diminished with repeated stimulations (Fig. 4C). Trafficking-associated cytokines, including MCP-1 and RANTES, were likewise upregulated in day 7 products (Fig. 4D). Collectively, these findings demonstrate that both manufacturing timelines generate functionally active CAR T-cells capable of robust cytokine secretion upon antigen encounter, with day 7 products exhibiting a more pronounced secretion profile across multiple cytokine classes. A full list and results of the entire 48-plex of cytokines tested is available in Supplemental Table 2.

### Gene expression analysis

**Fig. 5:**
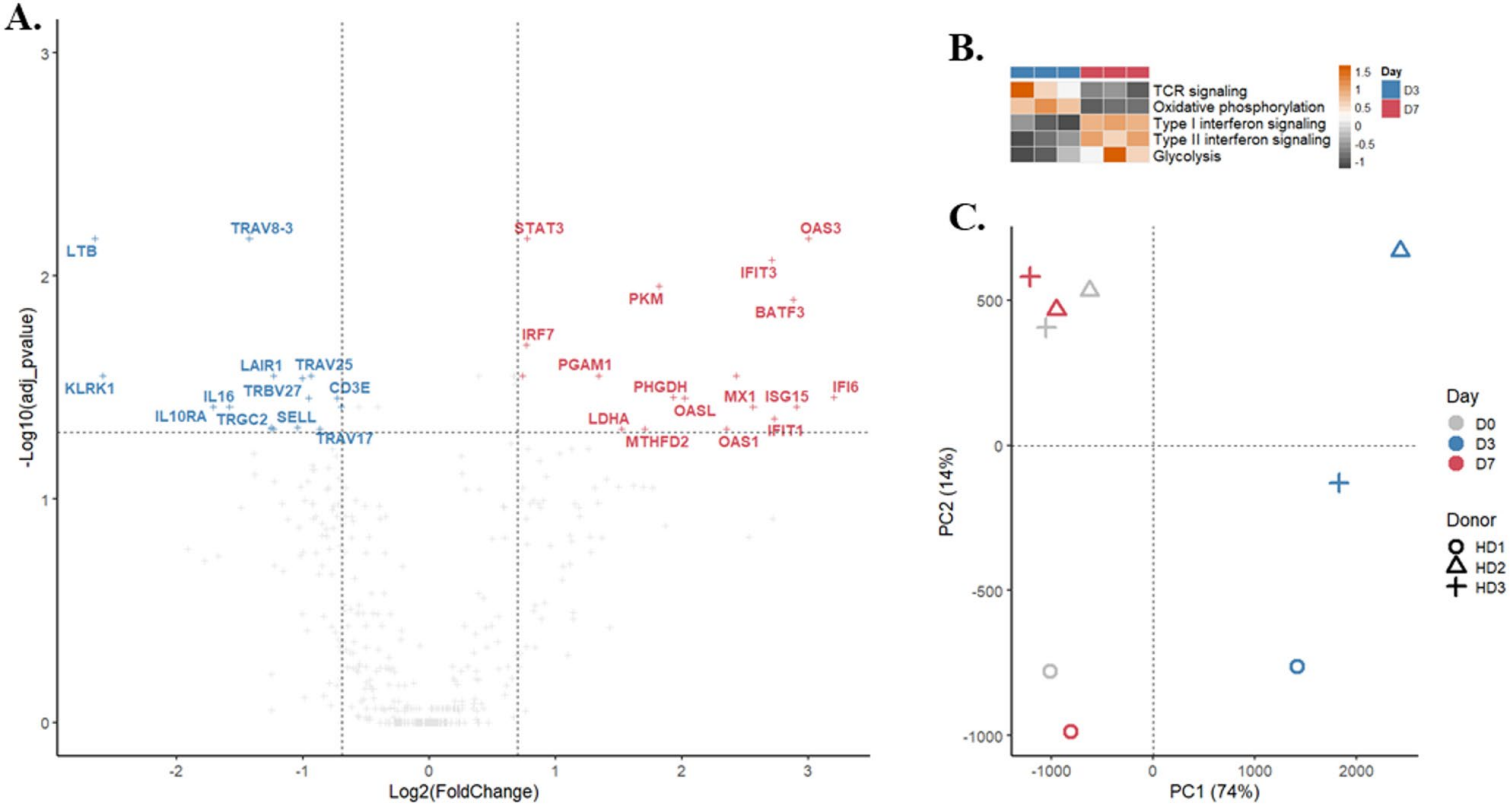
Comparison of gene expression and functional profiles between 3-day (early) and 7-day (late) CAR T-cell Products. **(A)** Differential gene expression analysis between 3-day and 7-day products. Genes significantly upregulated on day 7 products (red) include classic interferon-stimulated genes (e.g., *IFIT1*, *OAS3*), glycolytic enzymes (*LDHA*, *PGAM1*), and transcription factors associated with effector differentiation (*BATF3*, *STAT3*). Genes upregulated in day 3 products (blue) include memory-associated and TCR signaling genes (e.g., *SELL*, *TRAV3*, *LTB*), suggesting a less differentiated phenotype. **(B)** Gene set enrichment analysis shows increased TCR signaling, glycolysis, oxidative phosphorylation, and both type I and II interferon signaling pathways on day 7 products compared to day 3 (adjusted p-values indicated: *p* < 0.05; *p* < 0.01). Each row represents a gene set, and each column a sample; color scale indicates Z-score. **(C)** Principal component analysis (PCA) of global gene expression profiles separates samples based on culture duration (days 0, 3, and 7), with distinct clustering of day 3 and day 7 products regardless of donor (Healthy donor (HD)1–3). PC1 (74%) and PC2 (14%) account for the majority of variance

Gene expression analysis revealed distinct differences between the day 3 and day 7 CAR T-cell products (Fig. 5). The day 3 product expressed genes associated with a less differentiated, memory-like phenotype. These included multiple TCR-related genes (TRAV8-3, TRAV25, TRAV17, TRBV27, TRGC2, and CD3E), indicative of functional TCR signaling [22]. Elevated expression of SELL (CD62L), IL10RA, and IL-16 is consistent with a naïve or central memory phenotype and suggests involvement in early immune activation [23]. IL-16 expression, in particular, supports this early activation profile and is further corroborated by the cytokine secretion data [24]. The presence of LAIR1 may indicate the incorporation of regulatory or inhibitory features within this cell population [25].

In contrast, the day 7 product expressed genes characteristic of highly activated, antiviral T-cells engaged in rapid response and proliferation following antigen and type I interferon stimulation. This group showed marked upregulation of several interferon-stimulated genes (ISGs), including IRF7, OAS1, OAS3, OASL, IFIT1, IFIT3, ISG15, and MX1, all of which contribute to inhibition of viral replication and modulation of immune responses [26–29]. Increased glycolytic activity was suggested by elevated expression of PKM, PGAM1, and LDHA [30]. Furthermore, expression of STAT3 and BATF3 was consistent with differentiation toward Th17 cells and effector memory CD8^+^ T-cells, respectively [31].

These findings suggest that the day 3 product retains a memory-oriented, less differentiated profile with early activation potential, whereas the day 7 product exhibits a more terminally differentiated, highly activated phenotype optimized for immediate effector function.

## Discussion

This study demonstrated the feasibility of a rapid, 3-day CAR T-cell manufacturing process using whole blood as the starting material and a novel negative selection platform (T-isopure) to isolate T-cells while maintaining excellent viability, transduction efficiency, and functional activity. The combined use of whole blood input and an accelerated production timeline provide distinct advantages, particularly in the context of decentralized or point-of-care CAR T-cell therapy.

Whole blood is a more accessible and less resource-intensive source of T-cells compared to traditional leukapheresis products. We previously reported a 7-day manufacturing process starting with PBMCs isolated from whole blood using Ficoll density gradient separation [13]. Here, we advance this approach by introducing a negative T-cell selection platform and reducing the manufacturing timeline to just 3 days. Importantly, T-cell purification may broaden patient access, particularly for those with high numbers of circulating tumor cells or immunosuppressive monocytes, which may not be efficiently removed by Ficoll alone. Using the T-isopure antibody cocktail in combination with simple density gradient separation, we achieved efficient depletion of non-T-cell leukocyte populations, most notably granulocytes and B-cells, while isolating CD3^+^ T-cells with an average purity of 88.5%. Demonstrating this approach across 11 healthy donors and one pancreatic cancer patient represents an important first step in establishing the feasibility of using negatively selected T-cells from whole blood as a practical starting material for CAR T-cell manufacturing.

It has been previously reported that shortening manufacturing to 3 days from 9 days improved potency and persistence of CD19 CAR T-cells against NALM6 tumor cells in vivo [32]. Since then, an armored version of CD19 CAR T-cells secreting IL-18 using a similar 3-day process showed impressive clinical benefit in lymphoma patients relapsed from previous CD19 CAR T-cell treatment [33]. Elsewhere, others have generated CD19 CAR T-cells in < 48 h which yielded durable responses in leukemia patients [34–36]. Given the strong rationale for developing shortened manufacturing processes, we set out to design and characterize a manufacturing process that is fully GMP, focusing on key quality attributes including cell number, transduction, viability, and potency.

One of the major goals of this study was to compare the 3-day product to the same samples expanded under a traditional 7-day culture. Prior reports have suggested that abbreviated manufacturing timelines can better preserve early memory and stem-like T-cell subsets, which are thought to drive superior in vivo persistence and long-term antitumor activity [1, 37, 38]. It is important to note that the “3-day” and “7-day” products evaluated here were not generated using fundamentally distinct manufacturing workflows. Rather, the day 7 product reflects extended culture and expansion of the same initial manufacturing process used to generate the day 3 product. This design was intentionally chosen to minimize upstream procedural variability and isolate the impact of culture duration alone. However, this approach may limit direct comparison to more conventional clinical manufacturing platforms, including standard apheresis-derived products that incorporate additional process differences.

The phenotypic analysis did not reveal a statistically significant difference in stemness-associated surface markers between day 3 and day 7 products. Notably, the majority of our starting blood products already contained relatively high frequencies of T_scm_ and T_cm_ subsets compared with the more differentiated T_em_ population.

Functionally, the day 3 CAR T-cell product exhibited cytotoxic activity comparable to the day 7 product across all effector-to-target (E: T) ratios evaluated (5:1, 2:1, and 1:1) and under conditions of repeated stimulation. Both products effectively lysed target-expressing cells, with no statistically significant differences observed at any timepoint, including after multiple rounds of tumor cell challenge. Although the day 3 product yielded fewer total cells than the day 7 product, and functional comparisons performed at identical E: T ratios may not fully capture differences in potency on a per-cell basis or absolute cytotoxic capacity, the ability of day 3 CAR T cells to maintain equivalent cytotoxic activity at lower E: T ratios—including 1:1—suggests that the abbreviated manufacturing process does not impair intrinsic effector function. These findings support the conclusion that shortened culture duration preserves tumor-killing dynamics and functional fitness of CAR T cells. Nevertheless, additional studies evaluating threshold dosing or lower E: T ratios would be valuable to further define the functional limits and clinical dosing implications of this accelerated manufacturing approach.

To further characterize the functional differences between day 3 and day 7 CAR T-cell products, we evaluated cytokine secretion during repeated tumor re-stimulation. Day 7 products produced markedly higher levels of effector cytokines (IFN-γ, TNF-α, GM-CSF) and inflammatory/chemotactic mediators (RANTES, MIP-1β), consistent with a more activated and differentiated effector phenotype [39]. Day 7 products also secreted higher amounts of IL-2, IL-4, IL-10, and IL-15, cytokines often associated with proliferation, persistence, and regulatory function [40–42]. However, these increases likely reflect advanced differentiation rather than enhanced persistence potential. Persistence is better indicated by cellular state, specifically, a naïve or memory-like phenotype, rather than by baseline cytokine secretion, as less-differentiated T-cells typically remain more responsive to, rather than producers of, homeostatic cytokines such as IL-2 and IL-15 [10, 43].

Gene expression analysis further helped support this distinction. The day 3 product upregulated genes associated with naïve and central memory phenotypes including SELL (CD62L), IL10RA, and multiple TCR-associated transcripts. This suggests a less differentiated memory-like population with intact regulatory and homing capabilities [39, 44]. The day 7 product upregulated numerous interferon-stimulated genes, such as OAS1, IFIT1, and ISG15, and glycolytic enzymes (like PKM and LDHA), indicating a highly activated effector phenotype [45, 46]. Notably, the day 7 product also showed increased expression of canonical activation markers including CD69 and IL2RA (CD25), consistent with a more sustained activation state during extended culture. Prolonged activation and induction of interferon-stimulated gene programs during ex vivo expansion have been associated with increased differentiation and cellular stress responses, which may ultimately limit long-term persistence compared with less differentiated CAR T-cell populations. These transcriptomic differences complement the cytokine findings and support the hypothesis that the day 3 product may offer advantages in durability and stemness, while the day 7 product is more immediately cytotoxic and inflammatory. Altogether, the results from this study suggest that a rapid process may preserve valuable early differentiation states without sacrificing function.

Analysis of vector copy number (VCN) across culture duration demonstrated that values remained consistent and reliable between day 3 and day 7 harvests, with no significant drift toward higher or lower integration levels. Transduction efficiency remained relatively stable between day 3 and day 7; however, on day 3 the signal did not present as a well-defined peak and was therefore not readily quantifiable, whereas by day 7 it formed a distinct peak that was easily and reliably quantified. These observations are relevant when interpreting early timepoint CAR expression, as transient surface detection following transduction has been reported to reflect pseudotransduction artifacts that may overestimate true CAR expression. In the present study, however, the data do not support a substantial contribution of pseudotransduction. Such effects typically manifest as elevated early signals that decline over time, whereas our measurements showed stable or modestly increasing transduction efficiency from day 3 to day 7. Consistent with this, VCN measurements remained relatively stable across timepoints, with only minor fluctuations observed among individual samples and no systematic downward trend that would suggest loss of transiently detected CAR signal. Together, these findings indicate that early CAR detection at day 3 reflects stable vector integration rather than transient surface-associated expression and suggest that the abbreviated manufacturing process preserves transduction efficiency without compromising a key safety or quality attribute.

Despite these advantages, significant manufacturing and regulatory challenges remain. Although functionally active CAR T-cell products can be generated in only 3 days, current sterility assays require up to two weeks, delaying product release. This bottleneck diminishes the practical impact of rapid manufacturing unless paired with rapid-release testing strategies. Importantly, the successful generation of a functional product within 3 days should be viewed as an incremental step toward even more accelerated manufacturing paradigms that are under active investigation. A few groups have reported next-day or even < 24-hour manufacturing platforms capable of generating CAR T-cell products with preserved naïve or memory phenotypes and promising early efficacy signals in preclinical or early clinical studies [34, 47]. These efforts suggest that further compression of production timelines may be feasible with continued optimization strategies. Such advances would represent a paradigm shift for patients with rapidly progressive disease, where even short delays can compromise eligibility for therapy. Addressing both biological and regulatory constraints will therefore be essential to fully realize the clinical benefits of ultra-rapid, point-of-care CAR T-cell production.

The shortened manufacturing timeline is expected to reduce labor costs, as fewer technician hours are required for culture maintenance and in-process testing. In addition, reducing culture duration increases facility throughput, allowing more products to be manufactured within the same GMP footprint and time frame. This improved utilization of cleanroom space and equipment decreases the effective per-product facility cost, which is a major contributor to overall CAR T manufacturing expense.

Reagent use is also reduced with abbreviated culture, including decreased media consumption, cytokines, and single-use consumables. Although absolute reagent costs vary significantly across geographic regions and suppliers, fewer days in culture inherently translates to lower cumulative material use. Furthermore, the use of whole blood as a starting material eliminates the need for leukapheresis collection, which requires specialized equipment, trained personnel, extended chair time, and associated processing costs. Avoiding apheresis may therefore reduce both direct collection costs and logistical complexity, particularly in decentralized or resource-limited settings.

While formal cost-effectiveness analyses will be required to quantify these savings, the structural efficiencies inherent to accelerated manufacturing represent an important secondary advantage of this approach.

## Conclusion

This study demonstrates the technical feasibility and functional equivalence of a rapid, 3-day CAR T-cell manufacturing process using whole blood as a starting material. By isolating T-cells via negative selection and using a trispecific CD19-CD20-CD22 CAR and streamlined G-Rex culture, we produced viable, functional CAR T-cells without the need for leukapheresis or extended culture. These products maintained a favorable memory phenotype and gene expression, high viability, and potent in vitro cytotoxicity, all comparable to standard 7-day manufacturing. Importantly, this approach has the potential to reduce manufacturing costs, accelerate production timelines, and increase accessibility particularly in decentralized or resource-limited settings. Future work will focus on evaluating in vivo cytotoxicity, persistence, safety, and efficacy, as well as strategies for rapid release testing to enable broader clinical implementation.

## Supplementary Information

Below is the link to the electronic supplementary material.

Supplementary Material 1

Supplementary Material 2

Supplementary Material 3

## Data Availability

The data sets analyzed within the current study are available from the corresponding author on reasonable request.
