# Supplementary material for: Development of a 3-day manufacturing method to generate CD19-CD20-CD22 trispecific CAR T-cells from whole blood
Source: J Transl Med. 2026 Jul 15;24:923. doi: 10.1186/s12967-026-08306-8 (PMC13378159; doi:10.1186/s12967-026-08306-8)
Supplement: Supplementary file 1 — Supplementary Material 1 [file 12967_2026_8306_MOESM1_ESM.pdf]

Development of a 3-Day Manufacturing Method to generate CD19-CD20-CD22 Trispecific CAR T-cells from Whole Blood

Isabella Vignola[1], Michaela Prochazkova[1], Lipei Shao[1,] Tatyana Fuksenko[1], Ying Xiong[2], Zhongyu Zhu[2], Ibeawuchi Oparaocha[2], Oxana Slessareva[2], Megan Forrest[2], Rimas Orentas[2,3], Boro Dropulic[2], Ping Jin[1], Robert P. Somerville[1], David F. Stroncek[1], Hannah W. Song[1], Steven L. Highfill[1]

[1]Center for Cellular Engineering, Dept. of Transfusion Medicine, National Institutes of Health, Bethesda, MD
[2]Caring Cross, Gaithersburg, Maryland, USA
[3]The Johns Hopkins Bloomberg School of Public Health, Dept. Biochemistry and Molecular Biology, Baltimore MD

Supplementary Files

Sample	Donor 1	Donor 2	Donor 3	Donor 4	Donor 5	Donor 6	Donor 7	Donor 8	Donor 9	Donor 10	Donor 11	Patient
Age	40	35	48	70	72	25	64	79	62	52	29	71
Sex	F	M	F	F	M	F	M	M	M	F	F	F
Race*	2	3	1	1	1	3	2	1	1	1	3	1
Origin	CGT	CGT	NIH	NIH	NIH	NIH	NIH	NIH	NIH	CGT	CGT	CGT
Volume	294.2	276.2	263.1	250.2	206	393.3	258.4	266.5	315.9	131.5	342.4	34

* Race 1 = Caucasian ; 2 = African American; 3 = Asian

Supplemental Table 1. Donor Information Overview. Age, sex, race, where the sample was taken (origin) and volume of all 11 donors and 1 patient. Under origin, CGT indicates that the product was sourced from CGT global, shipped overnight and processed the next day, NIH indicates collection at NIH Clinical Center, where product was processed the same day it was donated.

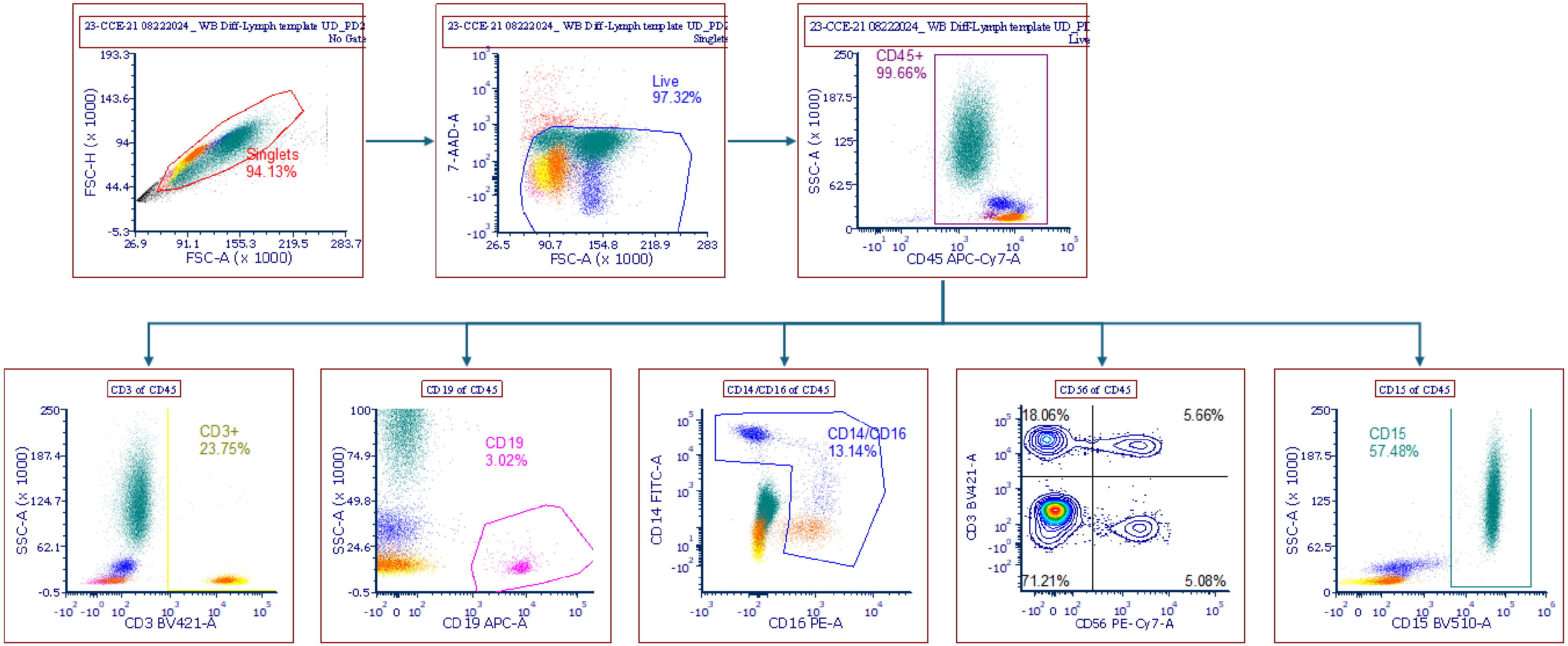

Supplementary Figure 1. Representative Flow Cytometry Gating. Cells were first gated on singlets, viable, and CD45. The CD45 group was then used to determine the relative percentages of CD3, CD19, CD14 and CD16, CD56, and CD15.

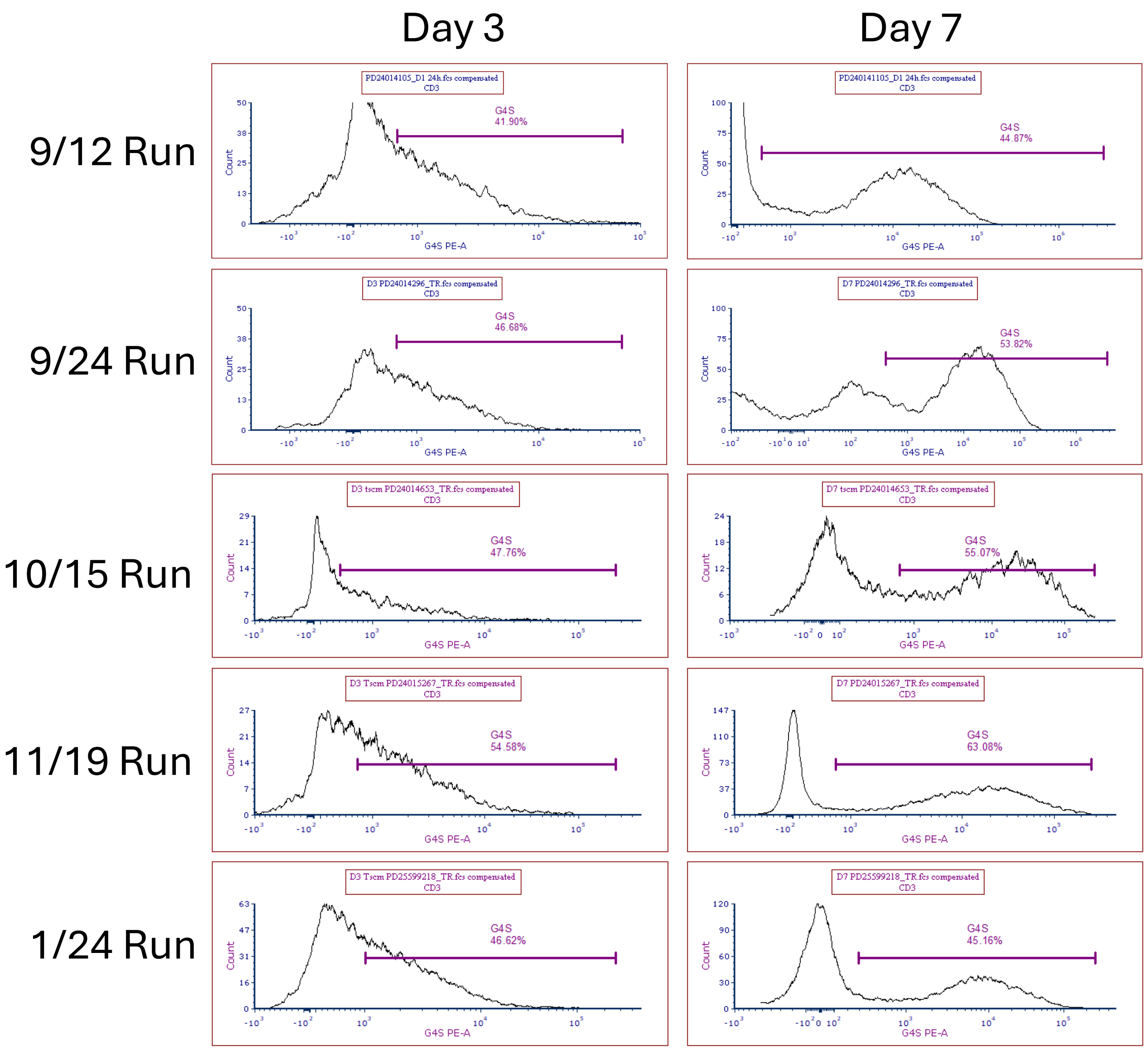

Supplementary Figure 2. Representative plots of G4S flow plots. Plots for the day 3 product are in the left column, plots from the day 7 product are in the right column.

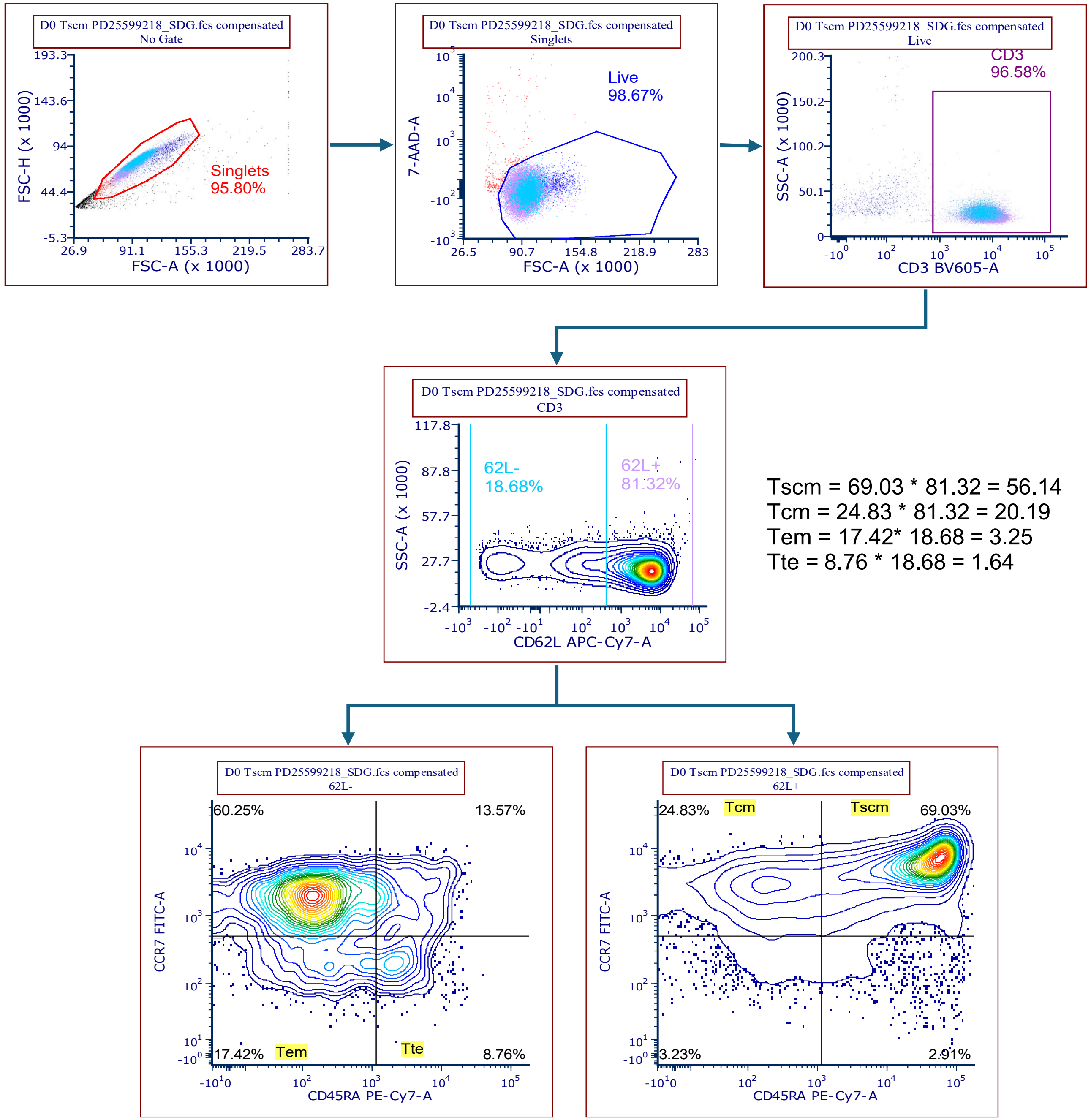

Supplementary Figure 3. Representative Flow Cytometry Gating for determining stemness. Cells were gated for singlets followed by live cells, CD3 cells, CD62L positive and negative, then on both CCR7 and CD45RA.

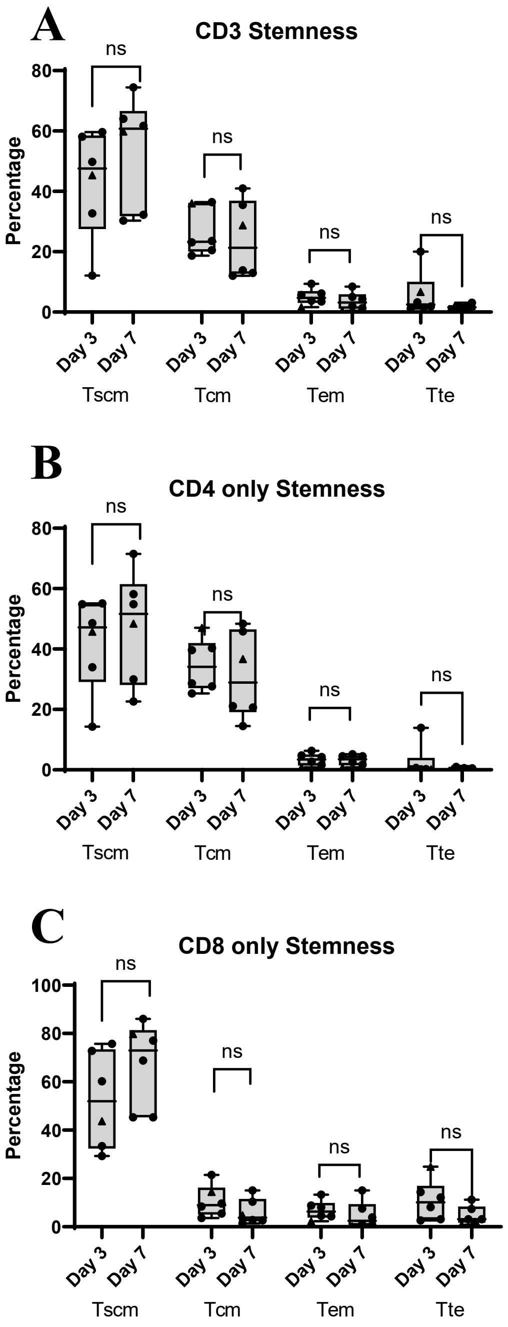

Supplementary Figure 4. Stemness, measured by flow with the different subsets defined as; Tscm/naive = CD62L+, CCR7+, CD45RA+, Tcm = CD62L+, CCR7+, CD45RA-, Tem = CD62L-, CCR7-, CD45RA-, Tte = CD62L-, CCR7-, CD45RA+, on days 3 and 7. (A) is of all CD3+ cells. (B) is CD4+ T-cells, (C) is CD8+ T-cells.
