# Supplementary material for: Development of a 3-day manufacturing method to generate CD19-CD20-CD22 trispecific CAR T-cells from whole blood
Source: J Transl Med. 2026 Jul 15;24:923. doi: 10.1186/s12967-026-08306-8 (PMC13378159; doi:10.1186/s12967-026-08306-8)
Supplement: Supplementary file 2 — Supplementary Material 2 [file 12967_2026_8306_MOESM2_ESM.pdf]

Supplemental Table 2.

Cytokine	Coculture Day	estimate	SE	p.value	Significance	DAY 3 product	DAY 7 product
b-NGF	DAY2	-13.90	6.01	0.043392746	*	44.77 ± 7.10	58.67 ± 4.73
	DAY4	-27.60	6.01	0.000995494	***	36.50 ± 13.43	64.10 ± 3.81
	DAY6	-20.00	7.36	0.021722144	*	30.90 ± 0.14	50.90 ± 2.69
CTACK	DAY2	-4.17	3.29	0.23446625	-	26.33 ± 4.07	30.50 ± 1.32
	DAY4	-14.60	3.29	0.001267302	**	20.50 ± 7.26	35.10 ± 2.82
	DAY6	-12.65	4.03	0.010574321	*	17.50 ± 1.41	30.15 ± 1.63
Eotaxin	DAY2	-12.40	23.69	0.612023224	-	112.03 ± 30.50	124.43 ± 17.43
	DAY4	-58.43	23.69	0.03327712	*	106.20 ± 49.12	164.63 ± 17.12
	DAY6	-59.75	29.01	0.066425171	-	104.30 ± 21.92	164.05 ± 7.42
FGF basic	DAY2	-49.07	16.16	0.012556234	*	120.60 ± 17.99	169.67 ± 9.36
	DAY4	-92.03	16.16	0.000199995	***	90.83 ± 33.86	182.87 ± 16.98
	DAY6	-63.15	19.80	0.009654511	**	71.75 ± 3.18	134.90 ± 14.71
G-CSF	DAY2	16.47	14.51	0.282801301	-	205.17 ± 19.73	188.70 ± 9.63
	DAY4	-47.63	14.51	0.008239148	**	176.00 ± 20.79	223.63 ± 16.75
	DAY6	-47.55	17.77	0.023238706	*	191.00 ± 27.58	238.55 ± 2.47
GM-CSF	DAY2	-3628.33	1582.12	0.044758847	*	15698.43 ± 1984.39	19326.77 ± 257.13
	DAY4	-6209.17	1582.12	0.002845096	**	15592.87 ± 3643.37	21802.03 ± 317.69
	DAY6	-5251.30	1937.69	0.021930626	*	14908.75 ± 1227.89	20160.05 ± 1131.72
GRO-a	DAY2	-96.20	49.61	0.081177244	-	355.80 ± 66.14	452.00 ± 36.68
	DAY4	-233.40	49.61	0.000835107	***	272.93 ± 97.77	506.33 ± 47.92
	DAY6	-242.75	60.75	0.002536355	**	236.15 ± 15.34	478.90 ± 39.03
HGF	DAY2	-18.30	8.07	0.046647794	*	43.80 ± 9.18	62.10 ± 6.42
	DAY4	-32.33	8.07	0.002481953	**	34.50 ± 16.80	66.83 ± 8.62
	DAY6	-16.60	9.88	0.123769703	-	28.65 ± 1.20	45.25 ± 3.18
IFN-a2	DAY2	-7.83	3.47	0.047734344	*	24.00 ± 4.00	31.83 ± 1.04
	DAY4	-15.40	3.47	0.001265924	**	19.43 ± 7.32	34.83 ± 4.25
	DAY6	-10.10	4.25	0.038978906	*	16.65 ± 0.49	26.75 ± 1.77
IFN-g	DAY2	-5502.53	2355.04	0.041587656	*	6878.67 ± 2461.56	12381.20 ± 3117.86
	DAY4	-8025.27	2355.04	0.006684107	**	4075.00 ± 2992.25	12100.27 ± 3625.27
	DAY6	-3342.75	2884.33	0.273408244	-	1912.40 ± 1081.02	5255.15 ± 2504.36
IL-1a	DAY2	-24.60	14.20	0.113921189	-	64.00 ± 18.03	88.60 ± 10.39
	DAY4	-46.07	14.20	0.008814709	**	49.20 ± 22.41	95.27 ± 22.75
	DAY6	-34.85	17.39	0.072952519	-	36.65 ± 4.74	71.50 ± 9.90
IL-1b	DAY2	-19.00	9.49	0.073086041	-	62.43 ± 12.30	81.43 ± 6.01
	DAY4	-40.00	9.49	0.001783634	**	44.77 ± 19.90	84.77 ± 9.12
	DAY6	-24.50	11.62	0.061216271	-	34.00 ± 2.12	58.50 ± 3.54

IL-1ra	DAY2	34.67	118.86	0.776512522	-	107.13 ± 66.44	72.47 ± 3.06
	DAY4	50.83	118.86	0.677958929	-	154.70 ± 109.20	103.87 ± 14.31
	DAY6	-139.25	145.58	0.361351697	-	252.65 ± 146.87	391.90 ± 396.55
IL-2	DAY2	-1947.27	746.82	0.026157157	*	772.10 ± 899.48	2719.37 ± 1506.41
	DAY4	-837.90	746.82	0.288099321	-	139.20 ± 145.04	977.10 ± 1040.84
	DAY6	-29.25	914.67	0.975118192	-	47.65 ± 13.65	76.90 ± 23.48
IL-2Ra	DAY2	415.93	365.55	0.281713424	-	1429.70 ± 415.49	1013.77 ± 144.06
	DAY4	-8.43	365.55	0.982047869	-	1937.43 ± 829.45	1945.87 ± 343.00
	DAY6	71.75	447.70	0.875864559	-	2057.00 ± 28.99	1985.25 ± 73.89
IL-3	DAY2	-2512.37	2451.15	0.329529655	-	6478.10 ± 3169.38	8990.47 ± 2714.04
	DAY4	-3226.80	2451.15	0.217398296	-	4640.20 ± 3037.08	7867.00 ± 2750.87
	DAY6	-2096.05	3002.03	0.500958517	-	3606.25 ± 2809.69	5702.30 ± 3717.97
IL-4	DAY2	-61.10	15.86	0.003203797	**	49.17 ± 7.51	110.27 ± 39.47
	DAY4	-41.10	15.86	0.026910519	*	35.60 ± 13.15	76.70 ± 9.42
	DAY6	-22.15	19.43	0.28082971	-	27.00 ± 0.71	49.15 ± 4.74
IL-5	DAY2	-18.43	13.53	0.203015943	-	104.33 ± 18.11	122.77 ± 9.77
	DAY4	-66.03	13.53	0.000642043	***	91.43 ± 27.50	157.47 ± 11.24
	DAY6	-49.00	16.57	0.014370316	*	95.00 ± 7.07	144.00 ± 9.19
IL-6	DAY2	304.90	171.47	0.10573496	-	425.83 ± 341.93	120.93 ± 39.23
	DAY4	9.27	171.47	0.957964532	-	305.03 ± 267.93	295.77 ± 136.64
	DAY6	5.50	210.00	0.979620793	-	199.15 ± 148.70	193.65 ± 32.74
IL-7	DAY2	-0.50	3.37	0.884833723	-	14.87 ± 4.01	15.37 ± 2.80
	DAY4	-8.30	3.37	0.033302024	*	13.80 ± 6.54	22.10 ± 3.55
	DAY6	-7.00	4.12	0.12026203	-	13.40 ± 2.97	20.40 ± 1.56
IL-8	DAY2	1703.07	1642.16	0.324121304	-	2396.87 ± 2668.35	693.80 ± 767.79
	DAY4	162.53	1642.16	0.923113808	-	2155.97 ± 2467.06	1993.43 ± 1979.37
	DAY6	48.40	2011.23	0.981274272	-	2130.05 ± 2182.49	2081.65 ± 509.33
IL-9	DAY2	-97.60	148.69	0.526395692	-	780.50 ± 183.77	878.10 ± 47.37
	DAY4	-350.50	148.69	0.040146107	*	686.27 ± 343.72	1036.77 ± 94.23
	DAY6	-300.75	182.11	0.129657496	-	552.65 ± 74.03	853.40 ± 9.76
IL-10	DAY2	-196.73	69.63	0.017998829	*	73.27 ± 20.93	270.00 ± 166.27
	DAY4	-115.90	69.63	0.127006091	-	37.10 ± 17.14	153.00 ± 89.25
	DAY6	-63.15	85.28	0.47604646	-	29.75 ± 6.72	92.90 ± 1.98
IL-12	DAY2	-6.13	6.87	0.380365122	-	24.30 ± 10.95	30.43 ± 9.92
	DAY4	-15.03	6.87	0.037904076	*	21.22 ± 12.01	36.25 ± 13.46
	DAY6	-10.78	8.42	0.211824437	-	18.95 ± 10.49	29.72 ± 14.58
IL-13	DAY2	269.70	4103.05	0.948887062	-	16850.97 ± 5672.27	16581.27 ± 3793.27

	DAY4	-6140.20	4103.05	0.165402451	-	17183.93 ± 7897.04	23324.13 ± 1543.26
	DAY6	-4188.75	5025.19	0.423996776	-	19029.15 ± 5169.87	23217.90 ± 1783.46
IL-15	DAY2	-17.93	6.69	0.023098042	*	46.27 ± 7.83	64.20 ± 5.11
	DAY4	-29.73	6.69	0.001248264	**	35.37 ± 11.64	65.10 ± 8.53
	DAY6	-23.85	8.20	0.015564406	*	30.80 ± 0.00	54.65 ± 8.98
IL-16	DAY2	-32.00	154.89	0.840464784	-	359.27 ± 77.14	391.27 ± 88.73
	DAY4	454.20	154.89	0.014976739	*	1029.70 ± 323.23	575.50 ± 111.85
	DAY6	1169.40	189.70	0.000106262	***	2333.65 ± 208.81	1164.25 ± 233.70
IL-17A	DAY2	-562.30	756.19	0.474228266	-	698.50 ± 809.14	1260.80 ± 1065.54
	DAY4	-673.30	756.19	0.394174981	-	492.37 ± 622.13	1165.67 ± 1295.76
	DAY6	-503.35	926.14	0.598695681	-	383.05 ± 421.08	886.40 ± 829.29
IL-18	DAY2	-7.60	5.50	0.197447577	-	34.33 ± 6.51	41.93 ± 2.50
	DAY4	-17.57	5.50	0.009631958	**	31.43 ± 11.60	49.00 ± 6.14
	DAY6	-16.75	6.74	0.032287916	*	28.40 ± 1.27	45.15 ± 3.32
IP-10	DAY2	1474.60	1499.53	0.348617003	-	2654.10 ± 2119.76	1179.50 ± 850.22
	DAY4	-68.23	1499.53	0.964602164	-	3005.70 ± 2655.51	3073.93 ± 2006.01
	DAY6	-678.10	1836.54	0.719657202	-	3428.30 ± 893.78	4106.40 ± 588.17
LIF	DAY2	200.63	122.84	0.133467155	-	405.97 ± 145.23	205.33 ± 33.51
	DAY4	31.10	122.84	0.805267474	-	436.37 ± 265.52	405.27 ± 45.90
	DAY6	63.45	150.45	0.682145411	-	391.50 ± 159.81	328.05 ± 105.71
M-CSF	DAY2	60.10	99.86	0.560656515	-	254.37 ± 90.88	194.27 ± 68.49
	DAY4	-93.73	99.86	0.370006987	-	365.53 ± 183.48	459.27 ± 165.67
	DAY6	-156.00	122.30	0.230937011	-	455.50 ± 33.94	611.50 ± 16.97
MCP-1	DAY2	-473.17	156.23	0.012708283	*	631.50 ± 183.70	1104.67 ± 192.33
	DAY4	-595.30	156.23	0.003427562	**	371.47 ± 222.16	966.77 ± 212.28
	DAY6	-287.50	191.35	0.163871008	-	183.25 ± 83.09	470.75 ± 170.06
MCP-3	DAY2	-1.50	3.57	0.683598163	-	27.33 ± 6.60	28.83 ± 1.89
	DAY4	-10.83	3.57	0.012656162	*	17.50 ± 6.26	28.33 ± 2.93
	DAY6	-6.75	4.38	0.154106555	-	13.75 ± 1.06	20.50 ± 0.71
MIF	DAY2	-297.33	118.41	0.030851837	*	2156.03 ± 125.02	2453.37 ± 193.56
	DAY4	-316.00	118.41	0.023541037	*	2216.37 ± 141.63	2532.37 ± 175.15
	DAY6	-132.60	145.02	0.382035832	-	2279.40 ± 10.47	2412.00 ± 50.20
MIG	DAY2	117.03	150.56	0.454978895	-	171.70 ± 210.14	54.67 ± 9.00
	DAY4	120.00	150.56	0.443962252	-	240.60 ± 321.72	120.60 ± 48.11
	DAY6	62.30	184.40	0.742463902	-	196.55 ± 199.76	134.25 ± 4.60
MIP-1a	DAY2	-350.93	1141.98	0.764916614	-	24152.10 ± 560.40	24503.03 ± 210.40
	DAY4	-2035.37	1141.98	0.105030345	-	22750.33 ± 2748.97	24785.70 ± 187.40

	DAY6	-4089.10	1398.63	0.015205195	*	20843.80 ± 1911.31	24932.90 ± 89.24
MIP-1b	DAY2	-1968.00	1452.73	0.205328759	-	8659.77 ± 1898.72	10627.77 ± 1215.41
	DAY4	-5655.20	1452.73	0.002995789	**	6661.60 ± 2476.99	12316.80 ± 1443.16
	DAY6	-5636.95	1779.22	0.010017977	*	5492.05 ± 1994.39	11129.00 ± 1038.03
PDGF-bb	DAY2	-3.50	3.53	0.344716189	-	29.93 ± 3.87	33.43 ± 2.57
	DAY4	-10.77	3.53	0.012235637	*	26.00 ± 8.19	36.77 ± 1.12
	DAY6	-9.35	4.32	0.055808411	-	22.65 ± 1.63	32.00 ± 2.12
RANTES	DAY2	385.90	1891.75	0.842451773	-	5043.50 ± 2957.80	4657.60 ± 2227.60
	DAY4	-6349.50	1891.75	0.007286077	**	3940.60 ± 3506.12	10290.10 ± 867.62
	DAY6	-8735.75	2316.91	0.003658914	**	2435.40 ± 135.20	11171.15 ± 386.58
SCF	DAY2	-9.40	32.00	0.774963276	-	173.03 ± 42.49	182.43 ± 26.32
	DAY4	-74.07	32.00	0.04317541	*	167.93 ± 66.50	242.00 ± 8.67
	DAY6	-55.35	39.19	0.18823627	-	173.65 ± 35.85	229.00 ± 9.19
SCGF-b	DAY2	-182.83	101.57	0.10205259	-	400.43 ± 86.00	583.27 ± 111.14
	DAY4	-63.00	101.57	0.548974843	-	579.93 ± 96.06	642.93 ± 190.14
	DAY6	196.00	124.40	0.146211569	-	584.40 ± 84.00	388.40 ± 132.09
SDF-1a	DAY2	-162.20	54.70	0.014154659	*	308.27 ± 61.82	470.47 ± 54.76
	DAY4	-233.83	54.70	0.001623754	**	210.33 ± 94.50	444.17 ± 67.48
	DAY6	-150.40	66.99	0.048577053	*	138.00 ± 16.97	288.40 ± 63.07
TNF-a	DAY2	-964.33	694.07	0.194872055	-	1388.87 ± 615.25	2353.20 ± 892.45
	DAY4	-1830.70	694.07	0.024834792	*	1218.27 ± 980.87	3048.97 ± 811.57
	DAY6	-824.25	850.06	0.355093946	-	823.00 ± 528.21	1647.25 ± 1164.25
TNF-b	DAY2	427.17	1209.17	0.731217317	-	4386.97 ± 1816.23	3959.80 ± 398.89
	DAY4	-3024.47	1209.17	0.031377929	*	2944.37 ± 1889.77	5968.83 ± 1429.54
	DAY6	-1197.35	1480.92	0.437605068	-	1823.55 ± 1200.31	3020.90 ± 1531.45
TRAIL	DAY2	161.50	89.35	0.100812451	-	328.87 ± 79.12	167.37 ± 60.60
	DAY4	29.00	89.35	0.75219662	-	398.10 ± 174.78	369.10 ± 107.13
	DAY6	-70.25	109.43	0.535338509	-	344.65 ± 73.75	414.90 ± 101.96
VEGF	DAY2	-13.23	8.33	0.143243547	-	67.43 ± 7.06	80.67 ± 8.40
	DAY4	-49.53	8.33	0.000141982	***	44.93 ± 6.21	94.47 ± 14.34
	DAY6	-33.35	10.20	0.008447819	**	48.40 ± 3.39	81.75 ± 17.32
