# Supplementary material for: Development of a 3-day manufacturing method to generate CD19-CD20-CD22 trispecific CAR T-cells from whole blood
Source: J Transl Med. 2026 Jul 15;24:923. doi: 10.1186/s12967-026-08306-8 (PMC13378159; doi:10.1186/s12967-026-08306-8)
Supplement: Supplementary file 3 — Supplementary Material 3 [file 12967_2026_8306_MOESM3_ESM.pdf]

Development of a 3-Day Manufacturing Method to generate CD19-CD20-CD22 Trispecific CAR T-cells from Whole Blood

Isabella Vignola[1], Michaela Prochazkova[1], Lipei Shao[1,] Tatyana Fuksenko[1], Sarmila Sarkar[1], Hong Lei[1], Ying Xiong[2], Yanping Xie[2], Ibeawuchi Oparaocha[2], Oxana Slessareva[2], Megan Forrest[2], Rimas Orentas[2,3], Zhongyu Zhu[2], Boro Dropulic[2], Ping Jin[1], Robert P. Somerville[1], David F. Stroncek[1], Hannah W. Song[1], Steven L. Highfill[1]

[1]Center for Cellular Engineering, Dept. of Transfusion Medicine, National Institutes of Health, Bethesda, MD
[2]Caring Cross, Gaithersburg, Maryland, USA
[3]The Johns Hopkins Bloomberg School of Public Health, Dept. Biochemistry and Molecular Biology, Baltimore MD

Supplementary Files – Revised 03/06/2026

Sample	Donor 1	Donor 2	Donor 3	Donor 4	Donor 5	Donor 6	Donor 7	Donor 8	Donor 9	Donor 10	Donor 11	Patient
Age	40	35	48	70	72	25	64	79	62	52	29	71
Sex	F	M	F	F	M	F	M	M	M	F	F	F
Race*	2	3	1	1	1	3	2	1	1	1	3	1
Origin	CGT	CGT	NIH	NIH	NIH	NIH	NIH	NIH	NIH	CGT	CGT	CGT[illegible]
Volume	294.2	276.2	263.1	250.2	206	393.3	258.4	266.5	315.9	131.5	342.4	34
Hematocrit	39.4	49	32.1	35	40.2	33.8	43.5	39.8	38.4	44.3	46.7	21.8
Platelet/mL	210	186	334	133	146	286	130	99	73	116	130	46
TNC $x10^9$	3.58	2.08	1.99	1.43	0.83	3.87	1.42	1.2	2.01	1.01	2.39	0.248

* Race 1 = Caucasian ; 2 = African American; 3 = Asian; TNC; total nucleated cells

Supplemental Table 1. Donor information overview. Age, sex, race, where the sample was taken (origin), volume, hematocrit, platelet count and TNC of all 11 donors and 1 patient. Under origin, CGT indicates that the product was sourced from CGT global, shipped overnight and processed the next day, NIH indicates collection at NIH Clinical Center, where product was processed the same day it was donated.

Marker	Conjugate	Catalog #	Clone
CD3	APC-H7	BD 641397	SK7
CD3	BV421	BD 563798	SK7
CD4	AF-700	BD 557922	RPA-T4
CD8	BV-510	BD 563919	SK1
CD14	FITC	BD 340682	MφP9
CD15	BV510	Biolegend 323028	W6D3
CD16	PE	BD 340705	B73.1
CD19	APC	BD 340722	SJ25C1
CD25	PE-Cy7	BD 557741	M-A251
CD45	APC-Cy7	BD 348805	2D1
CD45RA	BV421	BD 562885	HI100
CD56	PE-Cy7	BD 335791	NCAM16.2
CD62L	PE-Cy7	BD 565535	DREG56
G4S	PE	CellSig 38907	n/a
Viability	7-AAD	BD 559925	n/a

Supplemental Table 2. Manufacturer and clones of flow cytometry antibodies used in studies.

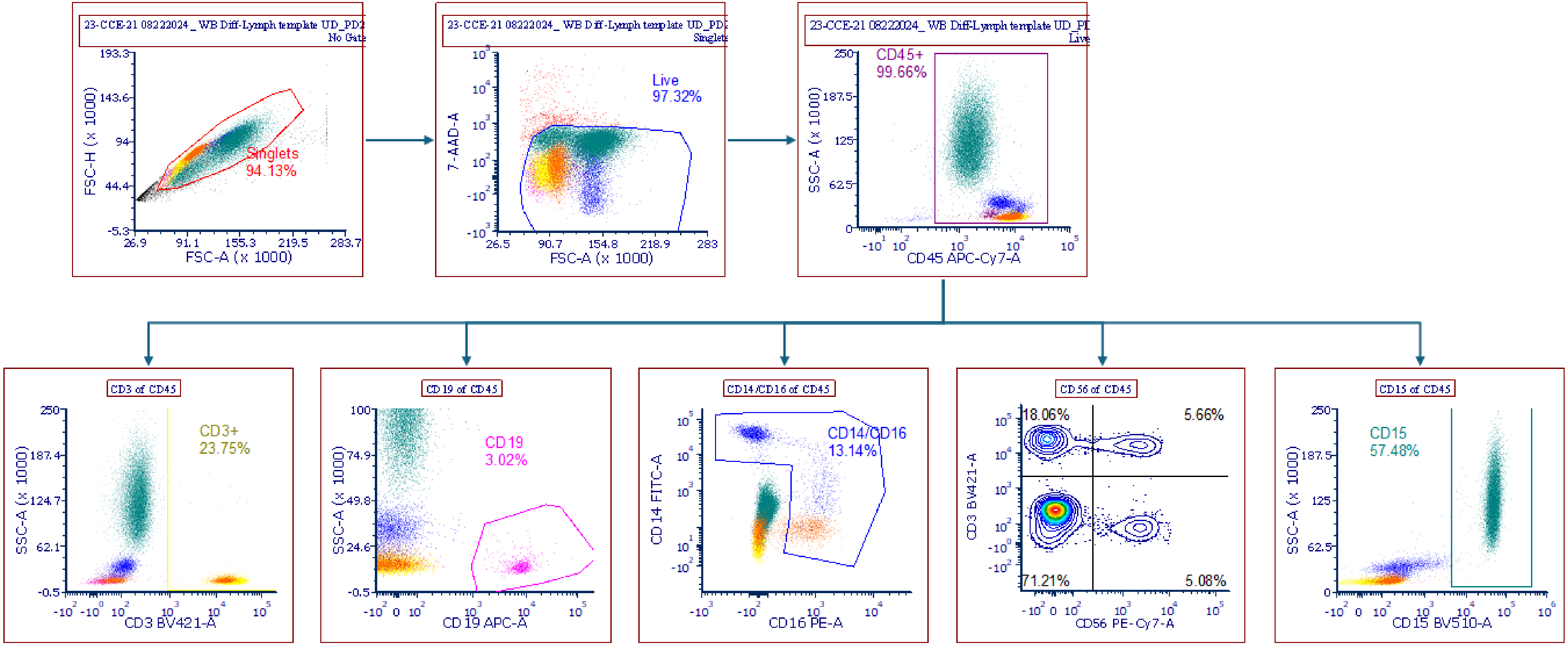

Supplementary Figure 1. Representative Flow Cytometry Gating. Cells were first gated on singlets, viable, and CD45. The CD45 group was then used to determine the relative percentages of CD3, CD19, CD14 and CD16, CD56, and CD15.

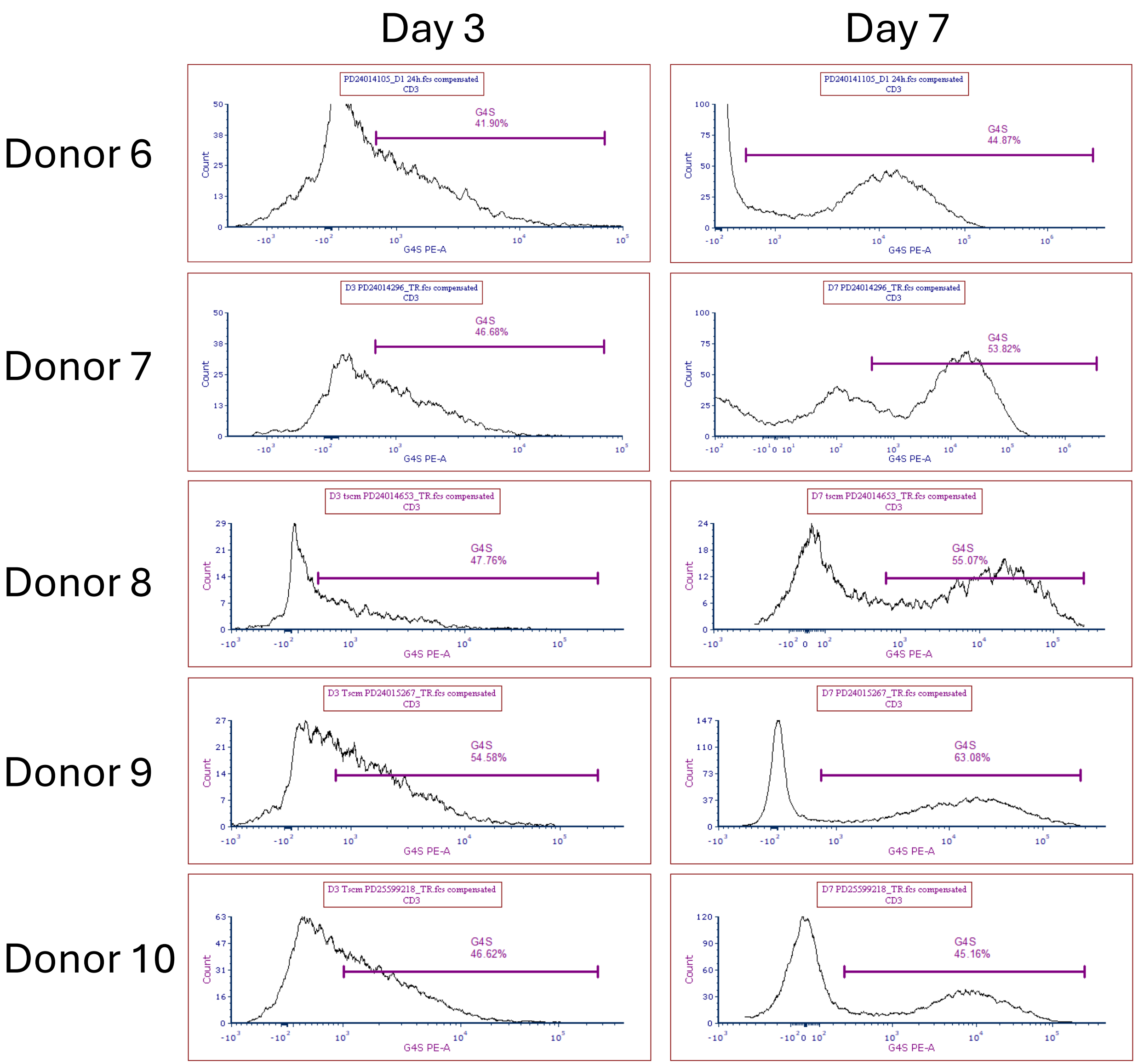

Supplementary Figure 2. Representative plots of G4S expression. Plots for the day 3 product are in the left column, plots from the day 7 product are in the right column.

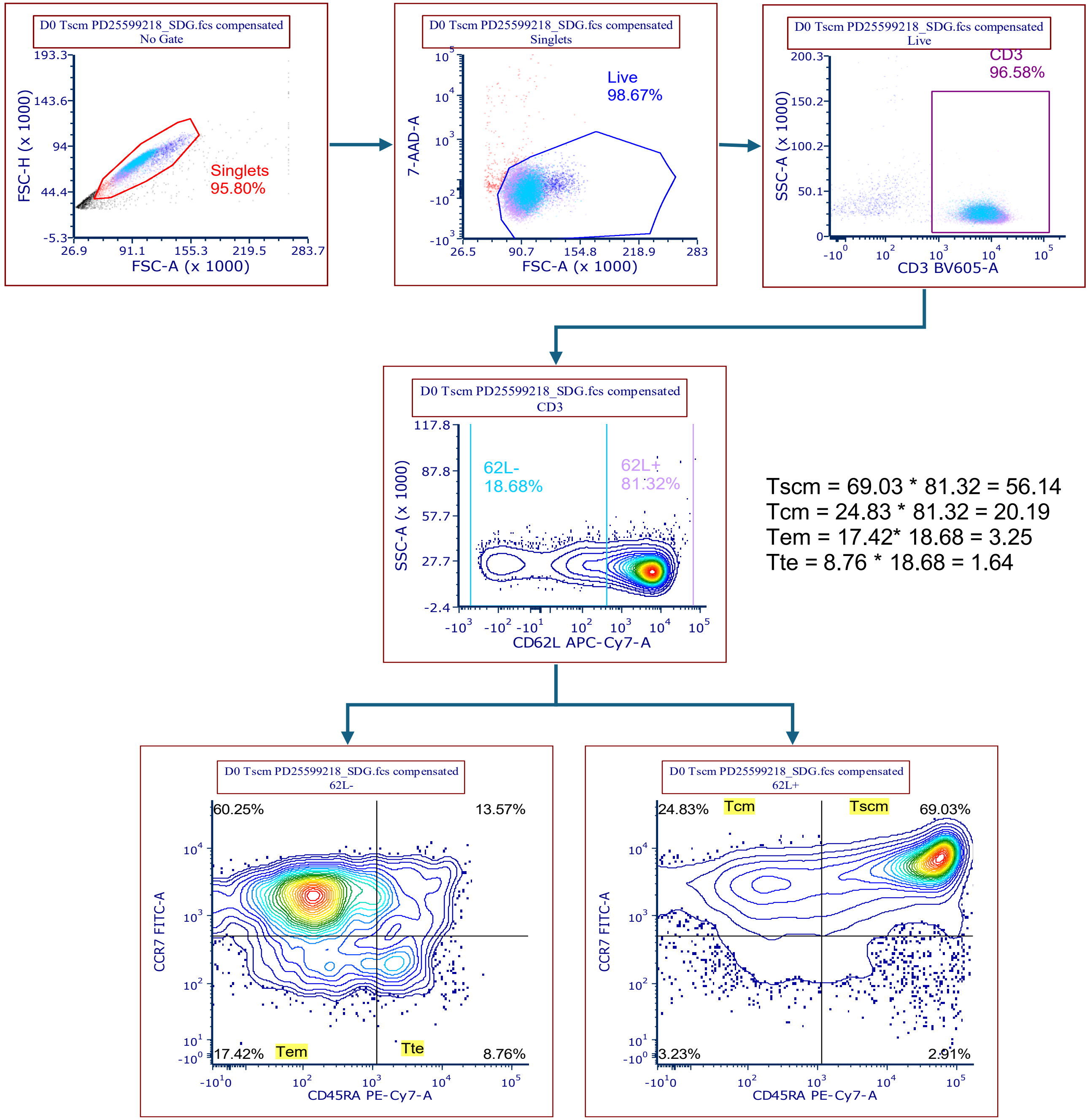

Supplementary Figure 3. Representative Flow Cytometry Gating for determining stemness. Cells were gated for singlets followed by live cells, CD3 cells, CD62L positive and negative, then on both CCR7 and CD45RA.
